# Adeno-associated virus vector modification based on directed evolution technology for gene therapy targeting head and neck squamous cell carcinoma

**DOI:** 10.3389/fonc.2025.1566584

**Published:** 2025-06-03

**Authors:** Yiyuan Zhu, Wei Ji, Qi Zhang, Yanbo Dong, Liangfa Liu

**Affiliations:** ^1^ Department of Otolaryngology and Head and Neck Surgery, Beijing Friendship Hospital, Capital Medical University, Beijing, China; ^2^ Department of Otolaryngology, Qilu Hospital of Shandong University, Jinan, Shandong, China; ^3^ Department of Cell Engineering, Beijing Institute of Biotechnology, Beijing, China

**Keywords:** AAV, gene therapy, head and neck squamous cell carcinoma, viral vector, molecular evolution

## Abstract

**Introduction:**

Adeno-associated virus (AAV) vectors are promising tools for cancer gene therapy, yet their clinical application in head and neck squamous cell carcinoma (HNSCC) is hindered by suboptimal transduction efficiency and off-target risks. Bioengineered AAV capsids require optimization to enhance tumor-specific targeting while minimizing systemic toxicity.

**Methods:**

We employed a directed evolution strategy combining DNA shuffling and site-directed mutagenesis to generate AAV variants. Five rounds of *in vitro* selection were performed using HNSCC cell lines (SCC-090, SCC-152, FaDu), followed by validation through *in vitro* transduction assays and *in vivo* studies in HNSCC xenograft mouse models. AAVzy9-3, a lead capsid variant, was further tested for α2δ1-targeted gene silencing efficacy.

**Results:**

This capsid demonstrated superior transduction efficiency in SCC-090, SCC-152 and FaDu cells when compared to the most efficient parental capsid. The validation of AAVzy9–3 targeting of HNSCC cells was validated through both *in vitro* and *in vivo* methods, employing a transplanted tumor mouse model. The results showed that AAVzy9–3 was more effective at infecting HNSCC cells than the wild type, while demonstrating reduced infectious potential toward other cells and organs. Additionally, the study used AAVzy9-3 to knockdown α2δ1 expression in a mouse model of HNSCC transplanted tumors, resulting in reduced tumor size.

**Discussion:**

The development of AAVzy9-3, a novel AAV variant with HNSCC-specific tumor tropism, addresses critical limitations of conventional AAVs. The *in vivo* antitumor activity validates its therapeutic potential for HNSCC.

## Introduction

Head and neck squamous cell carcinoma (HNSCC) ranks as the seventh most prevalent malignancy worldwide, accounting for approximately 4% of newly diagnosed cancers annually (1–4). Current therapeutic strategies for HNSCC encompass surgical resection, concurrent chemoradiotherapy, and emerging modalities such as targeted therapy and immunotherapy (5, 6). However, the clinical management of HNSCC remains challenging due to its high recurrence rate and propensity for metastasis (4). To address this, recent research has prioritized the eradication of residual tumor cells post-resection, with gene therapy emerging as a promising approach (7). Adeno-associated virus (AAV) vectors, known for their broad tissue tropism and low immunogenicity, have shown potential in transducing HNSCC cells (8). For instance, Haili Sun et al. (9) extended AAV expressing human FAS ligand significantly induces apoptosis and suppresses tumor growth in human laryngeal carcinoma cell line HEP-2, suggesting its potential as a gene therapy tool for HNSCC.

For cancer applications, AAV vectors have been used to deliver an extensive repertoire of transgenes in preclinical models (10). The AAV system is characterized by its remarkable versatility in the bioengineering of recombinant gene transfer vectors due to its low genetic complexity, which facilitates the cloning, packaging, and delivery of therapeutic gene expression cassettes (11). Minghong Jiang et al. (12) extended intratumoral delivery of AAV2/TRAIL in combination with cisplatin to a subcutaneous mouse model of HNSCC, resulting in 40% smaller tumors. However, these and other wild-type AAVs are suboptimal for HNSCC therapy due to their broad tropism and predilection for the liver.

Through the insertion of retargeting peptides and/or shuffling of the underlying *cap* genes and subsequent screening for a desired phenotype in cultured cells or *in vivo*, this directed evolution has been used to create synthetic AAV capsids with substantially novel properties, including the ability to evade pre-existing anti-AAV immunity or to transduce cells that are refractory to most or all wild-type AAV isolates (13). The advantage of the DNA shuffled library-based approach over other commonly used methods of AAV capsid modification is that it does not require detailed knowledge of the capsid structure and the structural effects of targeting ligand insertion (14).

The development of highly effective therapeutic strategies tailored to patients with HNSCC remains a pressing challenge (15). The calcium channel subunit α2δ1 encoded by *CACNA2D1*, is a critical component of L-type voltage-gated calcium channels (5). Breast cancer cells that are positive for α2δ1 mark tumor initiation, and α2δ1 is a potential functional tumor initial cells marker for breast cancer cells that further promote migration (16). α2δ1^+^ gastric cancer cells possessed cancer stem cells properties (17). Small-cell lung cancer cells expressing α2δ1 exhibit properties akin to cancer stem cells, which may contribute to chemoresistance (18). Targeting α2δ1 with specific antibodies significantly reduces tumor-initiating cells and has notable therapeutic effects on pancreatic cancer xenografts (19). In our previous studies, we observed that reducing the expression of α2δ1 reduced the tumorigenic capacity of HNSCC cells (20). These findings suggest that α2δ1 may serve as a promising target for anticancer therapies.

In this study we developed a new AAV library generated by DNA shuffling of *cap* genes from AAV1, 2, 3, 4, 5, 6, 7, 8, 9, rh10, 11 and 12, and induction of random point mutations, and then tested whether multiple rounds of selection in cultured human SCC-090 HNSCC cells would yield AAV capsids capable of efficiently transducing SCC-090 and other HNSCC cells. After five rounds of infection and rescue in SCC-090 cells, we isolated a new chimeric AAV capsid that was more efficient than the parental serotype (AAVrh10) in transducing HNSCC cells or tumors and less efficient in other cells and organs. We then we used AAVzy9–3 to knock down α2δ1 in a mouse model of HNSCC transplantation tumors with reduced tumor size.

## Materials and methods

### Cells

The FaDu human pharyngeal squamous cell carcinoma cell line was purchased from EallBio Biomedical Technology Co., Ltd. (Beijing, China, 06.0229). The human pharyngeal squamous cell carcinoma cell lines SCC-090 (IM-H654) and SCC-152 (IM-H586) were purchased from Xiamen Immocell Biotechnology Co., Ltd. (Xiamen Fujian, China). Human nasal epithelial cells (HNEpC) were purchased from Shanghai Enzyme-linked Biotechnology Co., Ltd. (Shanghai, China, ml-A396). Bronchial epithelium transformed with Ad12-SV40 2B (BEAS-2B) cell line was purchased from Wuhan Servicebio Technology Co., Ltd. (Wuhan Hubei, China, STCC10202P). HepG2 (CL-0103) and 293T (CL-0005) was purchased from Procell Life Science & Technology Co., Ltd. (Wuhan Hubei, China). FaDu were cultured in MEM (EallBio, 03.10001C) supplemented with 10% fetal bovine serum (Thermo Fisher Scientific, A5670801) and 1% penicillin/streptomycin (Thermo Fisher Scientific, 15140122), SCC-090, SCC-152, HNEpC, HepG2, and 293T cells were cultured in DMEM (EallBio, 03.PM1002A) supplemented with 10% FBS and 1% penicillin/streptomycin. These cells were cultivated at 37°C with 5% CO2, with medium renewal every 48 hours. To ensure the absence of mycoplasma, the cells were examined using the TransDetect^®^ PCR Mycoplasma Detection Kit (TransGen Biotech, FM321).

### AAV library construction

The *cap* genes for AAV serotypes AAV1, AAV2, AAV3, AAV4, AAV5, AAV6, AAV7, AAV8, AAV9, AAVrh10, AAV11 and AAV12 (twelve parental serotype capsid plasmids were custom-synthesized by Bomeide Biologics Co., Ltd.) were amplified by PCR using KeyPo Hi-Fidelity PCR Enzyme (Nanjing Vazyme Biotech, PK511-01/02/03). The PCR products of the *cap* genes were pooled and digested with DnaseI (New England Biolabs, M0303S), and the reaction was terminated with EDTA. A gel slice containing fragments between approximately 100 bp – 1000bp was excised. These fragments were reassembled into full-length chimeric *cap* based on partial homology using a primerless PCR. One microliter of the template from this PCR was used for a second PCR with a primer set (forward: 5′ GCATCTTTGAACAATAAATGACTAGTATG 3′, reverse: 5′ GGTTCCTGCGGCCGCTTA 3′), which binds to a conserved region of the full-length PCR product and contains SpeI and NotI restriction sites. The mutated gene libraries were generated by PCR using the above primers and a random point mutation kit (Beyotime, D0219M). The approximately 2.2kb PCR product was gel extracted and digested overnight with SpeI-HF and NotI-HF (both from New England Biolabs, R3133L and R3189L, respectively). The digested fragment was ligated into a similarly digested, replication competent AAV plasmid containing AAV2 rep and ITRs. The construct was transformed into DH10B cells (Beijing Tsingke Biotech, TSC-C09). Plasmid libraries were extracted using the PureLink HiPure Plasmid Maxiprep Kit (Invitrogen, K210007).

### AAV production

All AAVs in the present study were produced in 293T cells cultured adherently using the triple transfection protocol. This process involved the use of an AAV capsid plasmid, pPLUS^®^ AAV-Helper (Polyplus, 101000183), and an ITR plasmid containing the gene for enhanced green fluorescent protein (eGFP) under the control of a CMV promoter. The three plasmids were co-transfected using FectoVIR^®^-AAV (Polyplus, 101000044) at an equimolar ratio of 1:1:1. A total of 10^4^ 239T cells were used for transfection. Seventy-two hours post-transfection, the cells were harvested, and the virus was purified by preformed gradient of 15%, 25%, 40%, 60% iodixanol (OptiPrep, 1893) gradient centrifugation (21). The vector titer (copies/ml) was subsequently determined by quantitative PCR (qPCR) after alkaline lysis of the AAV particles to release the packaged vector genomes. The libraries were titrated using the primers (forward: 5′ TTCGATCAACTACGCAGACAG 3′ and reverse: 5’ GTCCGTGAGTGAAGCAGATAT 3′). qPCR quantification using a linearized plasmid standard (10²–10^10^ copies/µL).

### Selection of AAV libraries *in vitro*

SCC-090 cells were seeded at 5×10^5^ cells/well in a 6-well plate and incubated at 37°C. The medium was then replaced with serum-free DMEM, and AAV variant library virus was added at an MOI of 10^4^. Cells were incubated at 37°C for 4 hours. Uninternalized vector was eliminated by rinsing the cells twice with serum-free media, followed by the addition of wild-type Adenovirus serotype 5 (Ad5) at an MOI of 0.5 in complete media. On day 3 after incubation at 37°C, cells and supernatant were collected, subjected to three freeze-thaw cycles, and incubated at 60°C for 30 minutes to inactivate Ad5. Cell debris was removed by centrifugation, and the supernatant was collected for titration of total AAV genomes in crude cell lysate. The supernatant was then used for subsequent rounds of screening. After the fifth round, viral DNA was extracted using the Viral Genome Extraction Kit, and the full-length capsid gene was amplified from 2μl of viral DNA template using PCR enzyme and ligated to the AAV2 rep vector plasmid. The vector was transformed into DH10B bacterial cells, and a single clone was selected. The selected capsid gene was sequenced, and the partial sequences were assembled into full-length *cap* sequences and aligned with parental DNA sequences using SnapGene. Sequences were assigned different colors for visualization using conditional formatting in Microsoft Excel and GraphPad Prism.

### *In vitro* transduction assays

In order to compare the infection of the parental AAVs, SCC-090, SCC-152, FaDu cells were plated at a density of 2 × 10^4^ cells per well in a 96-well optical bottom plate (Thermo Fisher Scientific, 165306). Twenty-four hours after seeding, the cells were infected with 4 × 10^8^ vg/well (MOI = 2 × 10^4^) of eGFP or luciferase expressing, expressing the serotypes AAV1, AAV2, AAV3, AAV4, AAV5, AAV6, AAV7, AAV8, AAV9, AAVrh10, AAV11 and AAV12. Three days after infection, fluorescence intensity of eGFP was detected in each well using a FLUOstar Omega Multimode Microplate Reader (BMG LABTECH GmbH, 165306). Three days after infection, luciferase activity was measured using the FLUOstar Omega Multimode Microplate Reader after the addition of luciferase substrate. To compare AAVrh10 and AAVzy9-3, fluorescence intensity of eGFP was measured after three days after infection. To determine the targeting ability of AAVzy9-3, SCC-090, SCC-152, FaDu cells were infected with 4 × 10^8^ vg/well (MOI = 2 × 10^4^) of eGFP expressing AAVrh10 and AAVzy9-3. SCC-090, SCC-152, FaDu, HNEpC, BEAS-2B, HepG2 and 293T cells were plated at a density of 2 × 10^4^ cells per well in a 96-well plate. The cells were infected with 8 × 10^8^ vg/well (MOI = 4 × 10^4^) of eGFP expressing AAVzy9-3, fluorescence intensity of eGFP was measured after three days of infection.

### *In vivo* transduction assays

*In vivo* experiments were performed using BALB/c-nu male mice (age 5 weeks, body weight 19–22 g), purchased from SPF (Beijing) Biotechnology Co., Ltd. (Beijing, China). Animal experiments were conducted in accordance with the NIH Guide for the Care and Use of Laboratory Animals, and the experimental protocols were approved by the Experimental Animal Ethics Committee of Beijing Friendship Hospital of Capital Medical University (Ethical Review Approval No. 2023-P2-045-01).

A total of 16 BALB/c-nu mice were randomly assigned to two groups: the SCC-090 group and SCC-152 group. 2 × 10^5^ cells expressing infrared fluorescent protein 713 (iRFP) were injected subcutaneously into the right axilla of each mouse. Following a six-day observation period, each group was randomly divided into two groups (n = 4): the AAVrh10/luc group and the AAVzy9-3/luc group, the AAV vectors expressing luciferase were provided by Shandong Vigene Biosciences Co., Ltd. (Shandong, China), AAV vectors were injected into the tail vein of mice at either 2.0 × 10^13^ vg/kg. The *In vivo* bioluminescence imaging system IVIS200 (Caliper Life Science, Hopkinton, MA, USA) was used to evaluate the transduction efficiency of different AAV vectors in the organs and tumors of mice. Tissue samples were obtained at two weeks post-injection. About 25 mg of tissue was harvested per organ with a DNeasy Blood & Tissue Kit (Qiagen, 69506), for DNA extraction. qPCR was employed to quantify AAV copies using synthesized primers targeting the luciferase gene (forward: 5′ TGAGTACTTCGAAATGTCCGTTC 3′, reverse: 5′ GTATTCAGCCCATATCGTTTCAT 3′) (22).

Each BALB/c-nu mouse was subcutaneously injected with SCC-090 (2 × 10^5^ cells), resulting in two groups (n = 4) for subsequent analysis: the PBS group and the AAVzy9-3/shα2δ1 (expressing iRFP) treatment group. The successful shRNA interference sequence for α2δ1 is 5′ GCAATGAAGTTGTCTACTACA 3′. Oligonucleotides were annealed and inserted into the shRNA expression vector to form AAVzy9-3/shα2δ1 (for a detailed description of the method, see **Supplementary Methods**). The AAV vectors were administered via the tail vein of mice at a dosage of 10^13^ vg or an equivalent volume of PBS in the PBS group. Tumor size was measured with vernier calipers, and the tumor volume was calculated using the ellipsoid volume calculation formula is used for volume estimation V(mm^3^) = π/6 × L × W × H, where L represents the longest diameter, W represents the longest diameter perpendicular to the longest axis, and H represents the height of the tumor. On day 14 post-treatment, the mice and tumors were photographed, and the tumors were then excised and weighed.

### Western blot in mouse tissues

Protein extraction from tissues was facilitated by a cell lysis solution, followed by quantification with a BCA assay kit (Thermo Fisher, A55860). Lysates were then resolved by 10% SDS-PAGE (Nanjing Vazyme Biotech, P0690) and blotted onto nitrocellulose membranes, which were incubated with a 5% milk solution for one hour at ambient temperature. This was followed by an overnight incubation at 4°C with specific primary antibodies (Novus Biologicals). Afterward, membranes were treated with HRP-conjugated secondary antibodies for two hours at room temperature. Chemiluminescent detection was performed with reagent (Thermo Fisher, 34580), and band imaging was captured using the ChemiDoc MP system (Bio-Rad).

### Statistics

Unpaired *t* test was used to compare two distinct groups, whereas a one-way analysis of variance (ANOVA) test was selected for analysis of several groups (*p*<0.05 was considered statistically significant). Results are expressed as means ± SEM.

## Results

### Selection of an AAV library *in vitro* yields a vector which efficiently transduces HNSCC cells

**Figure 1:**
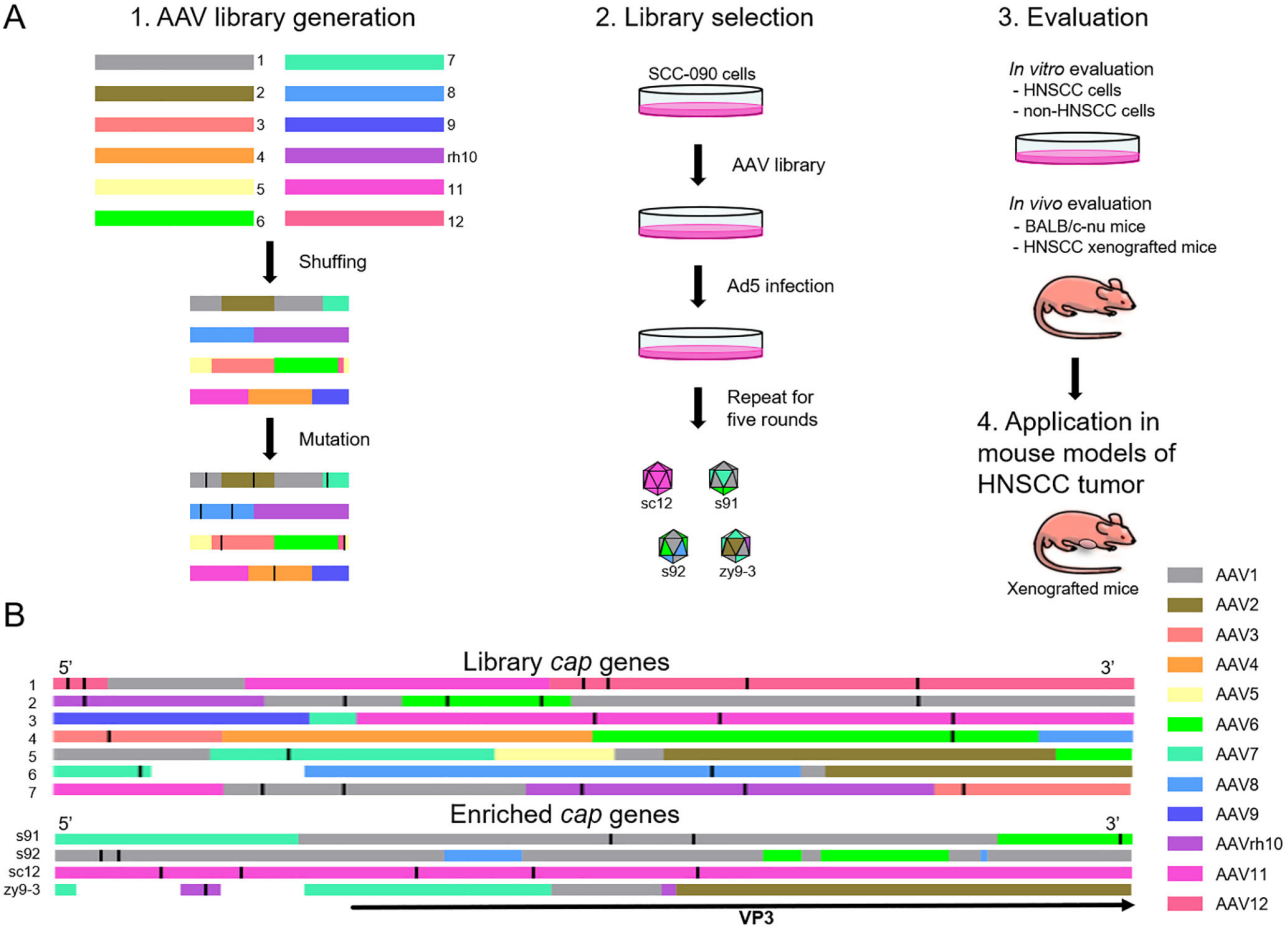
**(A)** Overview of AAV library generation, evolution, and validation. 1. Generation of AAV library through DNA shuffling and random mutation of multiple *cap* genes. 2. New variants through five rounds of screening cycles on SCC-090 cells *in vitro*. 3. Validation of vector specificity and targeting *in vivo* and *in vitro.* 4. Application of the new vector in a mouse model of transplanted HNSCC tumor. **(B)** Sequence analysis of AAV library, and the schematic shows the homology of each region of the *cap* gene. The different colors represent the various parental serotypes. The white sections of the sequences indicate that the parental source sequences are unknown due to the high number of mutations. The black straight line in each *cap* sequence represents a point mutation. The black arrowheads indicate the region of the sequence encoding the VP3 protein.

An overview of the workflow used in this study is shown in **Figure 1A**. Initially, we generated a recombinant capsid library by DNA family shuffling of AAV1, AAV2, AAV3, AAV4, AAV5, AAV6, AAV7, AAV8, AAV9, AAVrh10, AAV11 and AAV12 capsid genes, followed by random point mutations to finalize the library. We randomly selected seven capsid genes for representation (**Figure 1B**). Analysis revealed that the displayed capsid genes encode chimeric sequences, comprising two or more AAV serotypes, with one or more points mutations per sequence. After five rounds of *in vitro* screening in the SCC-090 cell line, four AAV variants were identified and sequenced (**Figure 1B**). Notably, AAVzy9–3 emerged with the highest prevalence among the screened capsids, suggesting superior infectivity in this cell line. The specific sequence of the *cap* gene of AAVzy9–3 is given in the **Supplementary Material**.

### Evaluation of parental capsids for HNSCC transduction efficiency

**Figure 2:**
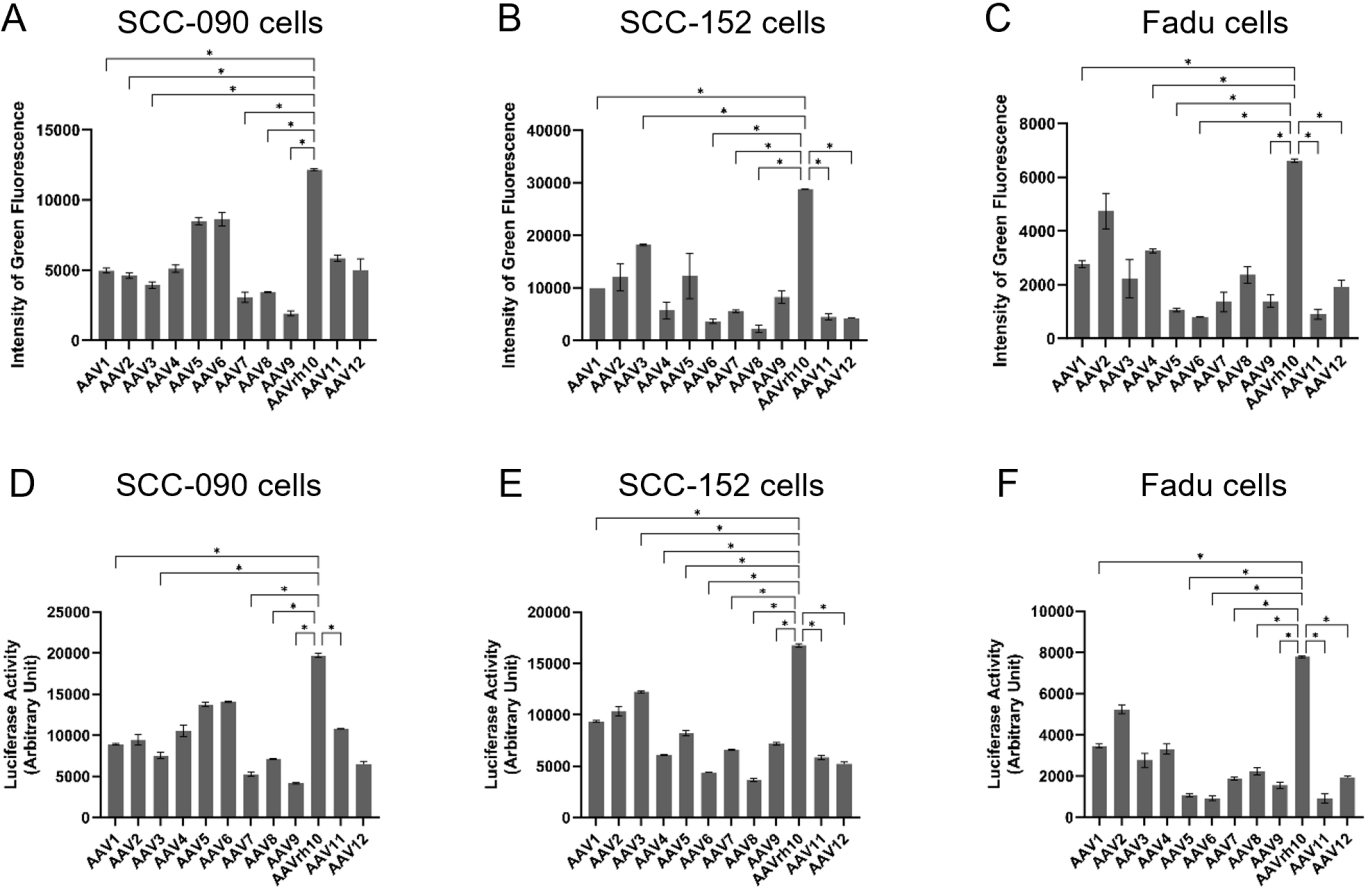
The transduction efficiency of AAV1, AAV2, AAV3, AAV4, AAV5, AAV6, AAV7, AAV8, AAV9, AAVrh10, AAV11 and AAV12 on head and neck squamous cell carcinoma (HNSCC) was evaluated. Three days post-infection with AAV vectors expressing enhanced green fluorescence protein (eGFP) at a multiplicity of infection (MOI) of 2 × 10^4^, the intensity of green fluorescence was quantified in **(A)** SCC-090, **(B)** SCC-152, and **(C)** FaDu cells (n = 4). Three days post-infection with AAV vectors expressing luciferase at a multiplicity of infection (MOI) of 2 × 10^4^, the activities of luciferase was quantified in **(D)** SCC-090, **(E)** SCC-152, and **(F)** FaDu cells (n = 4). * indicates P < 0.05.

The transduction efficiencies of AAV1, AAV2, AAV3, AAV4, AAV5, AAV6, AAV7, AAV8, AAV9, AAVrh10, AAV11 and AAV12 were assessed in three HNSCC cell lines: SCC-090, SCC-152 and FaDu cells. Following infection with an AAV vector expressing enhanced green fluorescent protein (eGFP) at an MOI of 2 × 10^4^ for 72 hours, AAVrh10 demonstrated the highest intensity of green fluorescence in SCC-090 cells (**Figure 2A**), indicating superior transduction efficiency. Similarly, AAVrh10 exhibited the highest transduction efficiency in SCC-152 and FaDu cells compared to other serotypes (**Figures 2B, C**). Similarly, after infecting cells with an AAV vector expressing luciferase at an MOI of 2 × 10^4^ for 72 hours, AAVrh10 showed the highest luciferase activity in SCC-090 cells (**Figure 2D**), SCC-152 (**Figure 2E**) and FaDu cells (**Figure 2F**).

### Assessment of transduction efficiency and tropism of several HNSCC cells by new AAV variant *in vitro*

**Figure 3:**
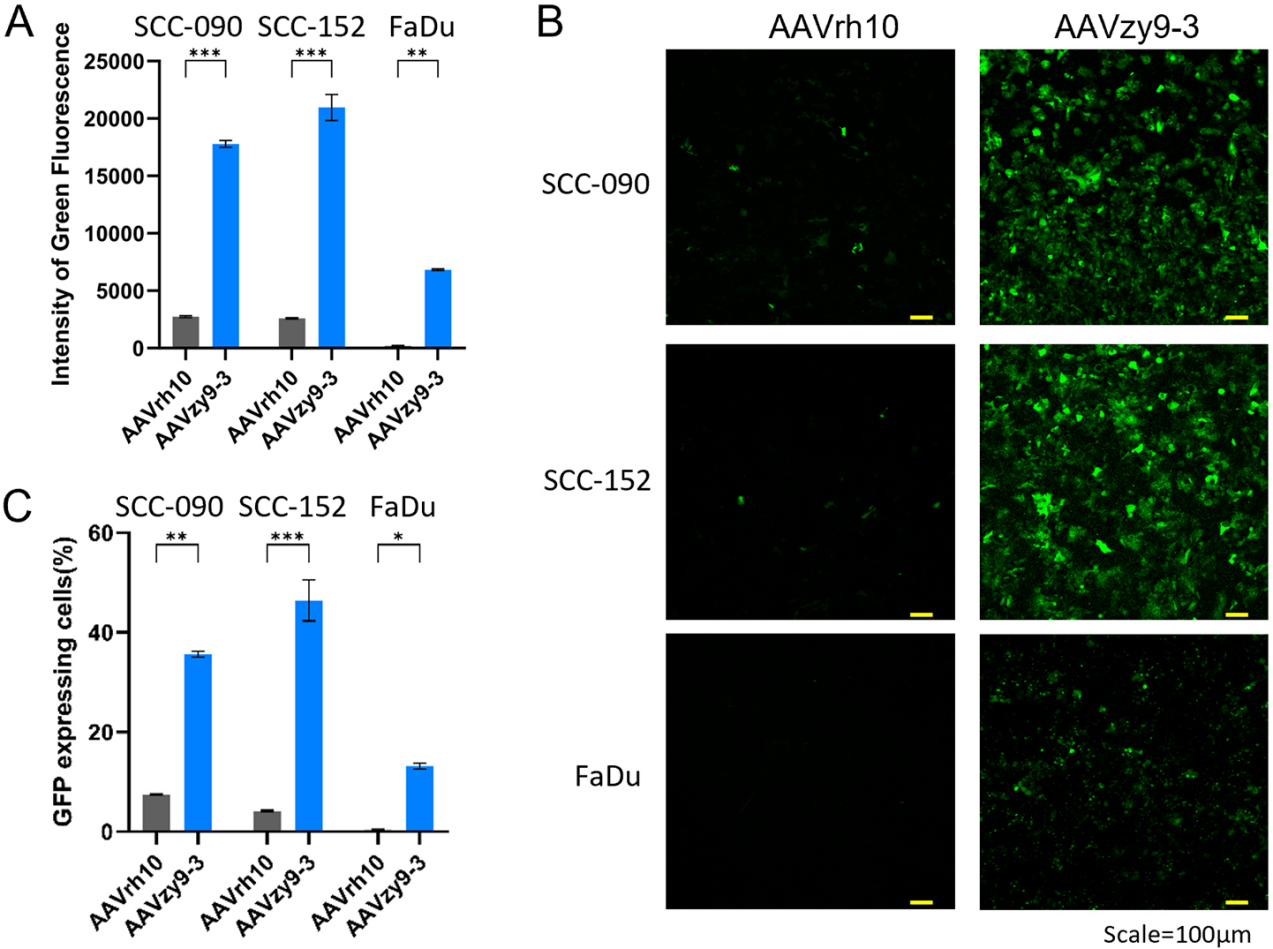
**(A)** Transduction efficiency of AAVzy9–3 and AAVrh10 in HNSCC cell lines SCC-090, SCC-152, and FaDu. Intensity of green fluorescence was measured 3 days post-infection with eGFP-expressing AAV vectors at MOI of 2×10^4^. **(B)** Representative fluorescence micrographs. Scale bar, 100 μm. **(C)** Percentage of cells expressing GFP. * indicates P < 0.05, ** indicates P < 0.01, *** indicates P < 0.001.

We compared the transduction efficiencies of AAVzy9–3 and AAVrh10, the latter being the most efficient parental serotype for infecting HNSCC cell lines (as shown in **Figure 2**), in three HNSCC cell lines: SCC-090, SCC-152 and FaDu. After infection with AAV vectors expressing eGFP at an MOI of 2×10^4^ for 3 days, AAVzy9–3 showed a significantly higher intensity of green fluorescence compared to AAVrh10 in all three cell lines. The fluorescence intensity induced by AAVzy9–3 were 18-fold, 20-fold, and 37-fold higher than those induced by AAVrh10 in SCC-090, SCC-152, and FaDu cells, respectively (**Figures 3A, C**). Representative fluorescence images are shown in **Figure 3B**.

**Figure 4:**
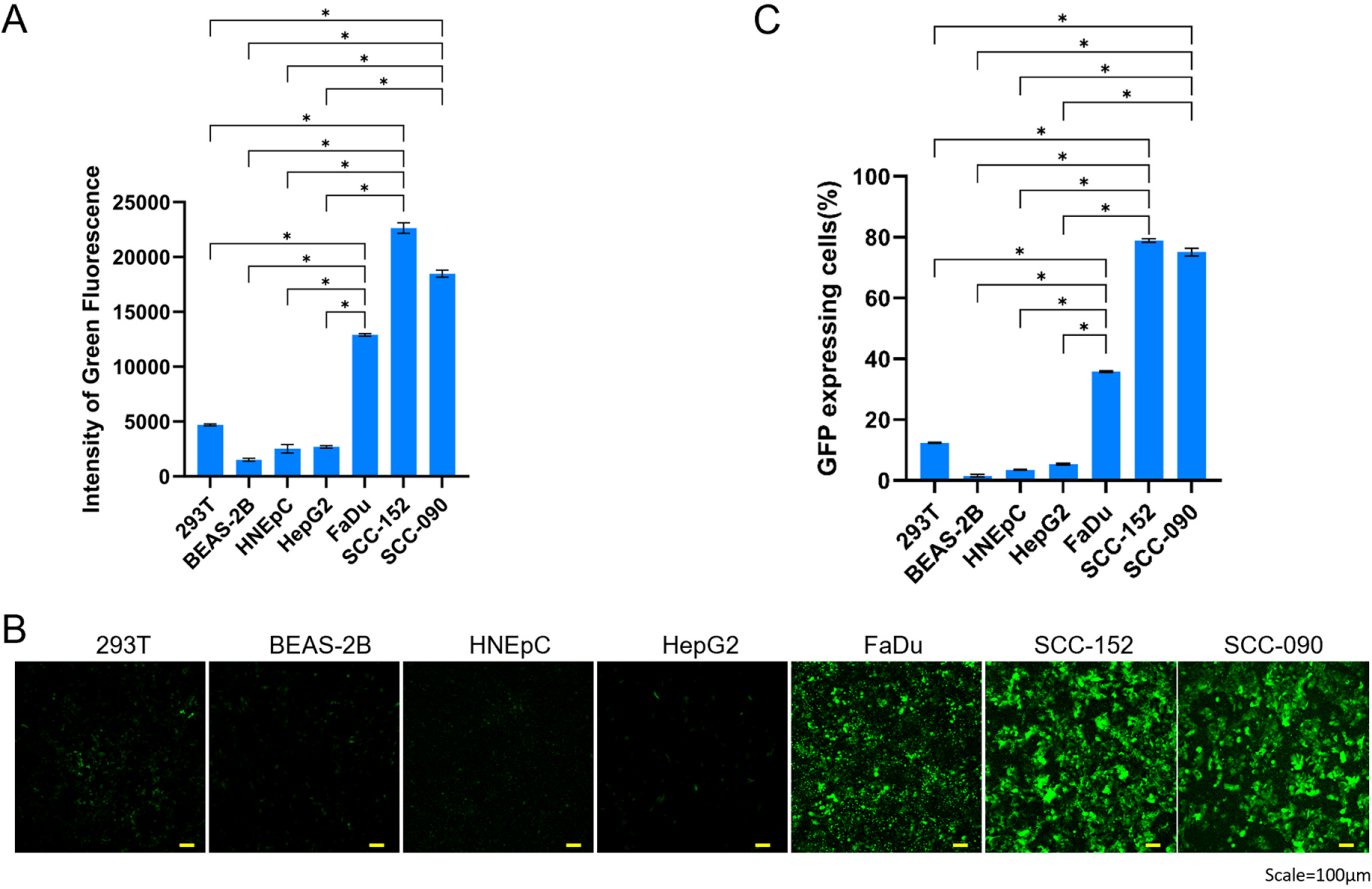
**(A)** Comparative transduction efficiency of AAVzy9–3 in HNSCC cell lines SCC-090, SCC-152, and FaDu, alongside additional cell lines 239T, BEAS-2B, and HNEpC. intensity of green fluorescence was measured 3 days post-infection with eGFP-expressing AAV vectors at an MOI of 4 × 10^4^. **(B)** Representative fluorescence micrographs. Scale bar, 100 μm. **(C)** Percentage of cells expressing GFP. * indicates P < 0.05.

To evaluate the targeting specificity of AAVzy9-3, we compared its transduction efficiency between HNSCC cell lines (SCC-090, SCC-152, FaDu) and other cell lines (293T, BEAS-2B, HNEpC). All cell lines were infected at an MOI of 4 × 10^4^ for AAVzy9-3, and green fluorescence intensity was measured 72 hours post-infection. The cell lines BEAS-2B and HNEpC are the normal mucosal epithelial cells of the head and neck. The aforementioned two cell comparisons can be considered to reflect the relationship between tumor and paraneoplastic. The data demonstrate that the intensity of green fluorescence in HNSCC cell lines was markedly elevated in comparison to other cell lines infected with AAVzy9-3 (**Figures 4A, C**). The corresponding fluorescence images are presented in **Figure 4B**, indicating that the capacity of AAVzy9–3 to infect the HNSCC cell lines is more pronounced than the other cell lines.

### *In vivo* assessment of transduction efficiency of a novel AAV variant in HNSCC tumors

**Figure 5:**
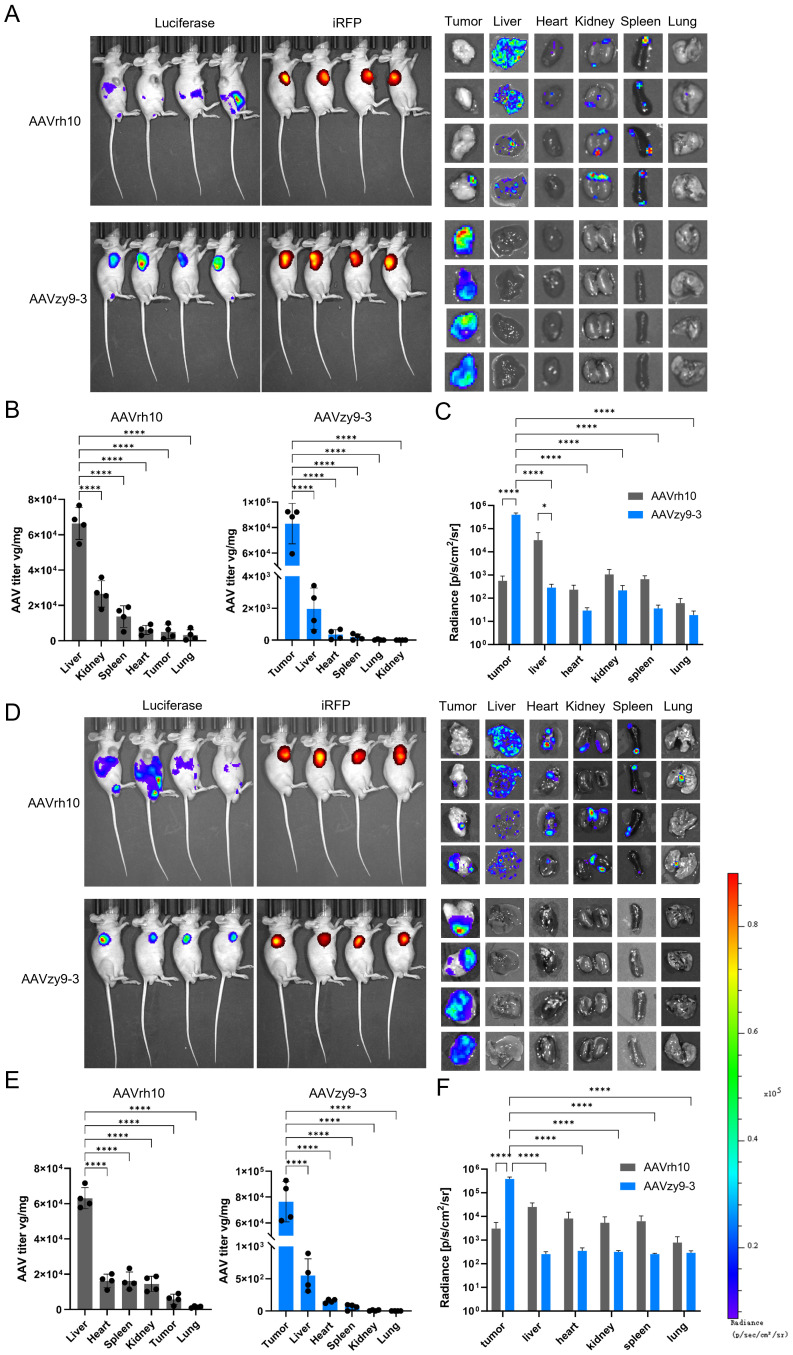
Nude mice (BALB/c-nu) were injected subcutaneously with 2 × 10^5^ expressing infrared fluorescent protein 713 (iRFP) SCC-090 or SCC-152 cells per axilla (n = 8). Six days following the injection, each mouse was administered an intravenous injection of 2×10^13^ vg/kg AAVzy9-3/luc or AAVrh10/luc (n = 4), and the mice were subsequently analyzed 14 days following the injection. **(A)** Whole-body bioluminescence imaging of SCC-090 xenografted mice was conducted two weeks following injection with the indicated vectors. Bioluminescence from the mouse iRFP and luciferase was then acquired, followed by luminescence imaging of isolated organs and tissues. **(B)** AAV copy numbers were determined by qPCR using primers specific for luciferase (means ± SEM, n = 4), and **(C)** regions of interest were delineated manually for individual tissues. The mean luciferase luminescence intensity was quantified for each tissue (means ± SEM, n = 4). **(D)** Similarly, whole-body bioluminescence imaging of SCC-152 xenografted mice was performed two weeks after vector injection, and **(E)** AAV copy numbers were assessed by qPCR using luciferase primers (means ± SEM, n = 4). Additionally, **(F)** manual region of interest analysis was applied to individual tissues. The mean luciferase luminescence intensity was calculated for each tissue (means ± SEM, n = 4). * indicates P < 0.05, **** indicates P < 0.0001.

To verify the transduction efficiency of AAVzy9–3 in HNSCC tumor-bearing mice, SCC-090 or SCC-152 cells expressing iRFP were injected subcutaneously into nude mice, and AAV encoding the luciferase gene (AAV/luc) was injected intravenously into the mice at 2 × 10^13^/kg 6 days later. *In vivo* imaging was performed 2 weeks after AAV injection and viral titers within individual tissues were examined (**Figure 5**). Interestingly, in both HNSCC tumor-bearing mice, mice injected with wild-type AAVrh10 exhibited strong intraperitoneal luciferase expression that essentially did not overlap with iRFP-expressing tumor tissues, and individual organ and tissue imaging showed luciferase expression in all tissues, with the strongest expression in the liver (**Figures 5A, C, D, F**), and the imaging results and detection of viral titers after extraction of the tissue genome extraction were consistent (**Figures 5B, E**). In contrast, AAVzy9-3-injected mice showed no luciferase expression in the peritoneal cavity and only a small amount of luciferase expression was seen in the testis of few mice, and all mice showed strong luciferase expression in iRFP-expressing tumors, and only tumor tissues showed luciferase expression on imaging of individual organs and tissues (**Figures 5A, D**), which were generally consistent with the viral titers detected by extracting the genome from the tissue genome. The imaging results and the detection of viral titers after extraction of the tissue genomes were generally consistent, with some organs having small amounts of virus detected in the tissue genomes despite the absence of luciferase (**Figures 5B, C, E, F**).

### Use of AAVzy9–3 vectors for gene therapy of HNSCC

**Figure 6:**
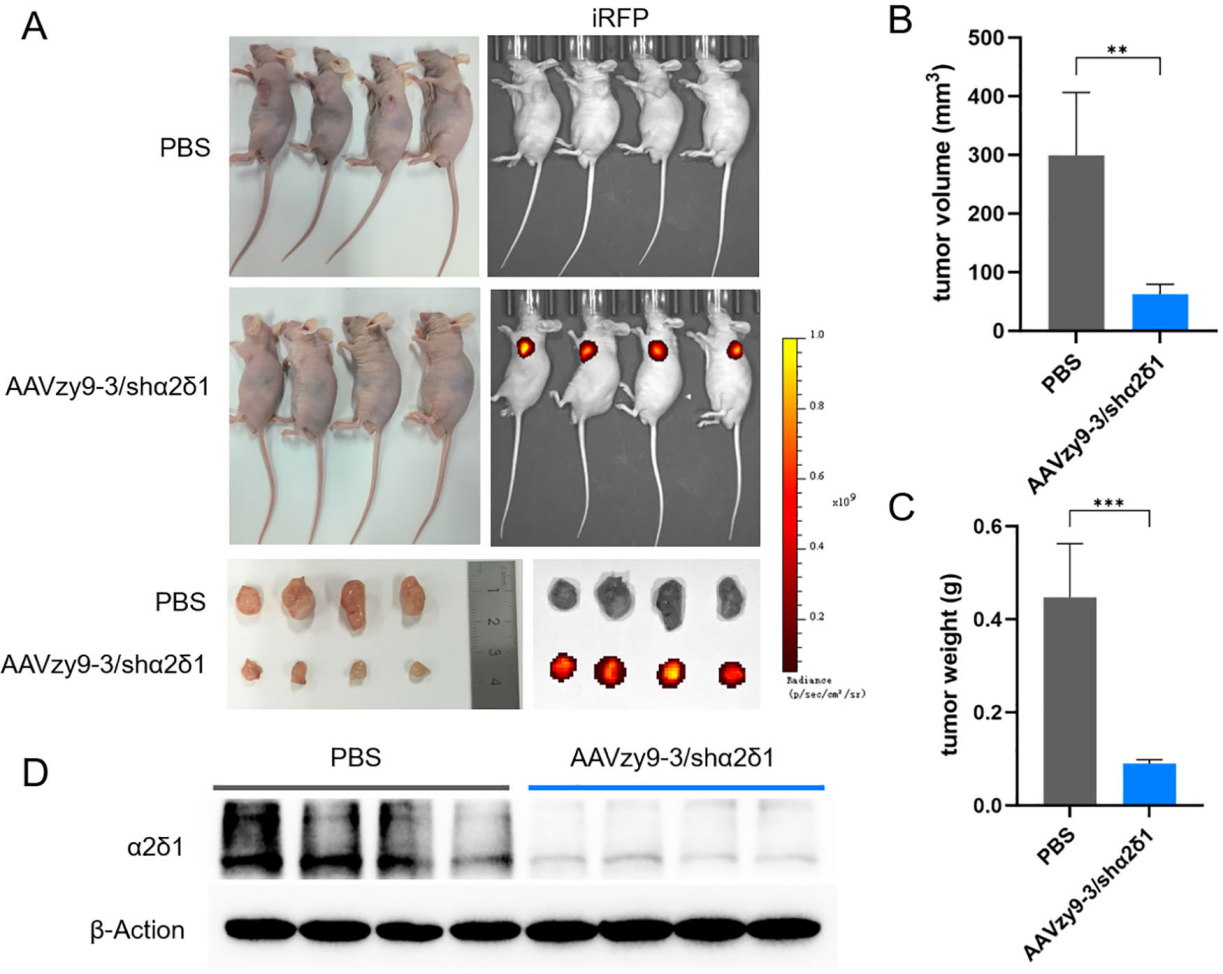
**(A)** Nude mice were subcutaneously inoculated with 2 × 10^5^ SCC-090 cells to form tumors. Subsequently, an intravenous injection of phosphate buffered saline (PBS) or AAVzy9-3/shα2δ1 (expressing iRFP) was administered after a period of six days. Tumor growth in mice at 20 days post-inoculation. The following images illustrate the expression of iRFP in mice. The following images illustrate the anatomical structure of mouse tumors and the expression of iRFP. **(B)** Tumor volume in mice. **(C)** Tumor weight in mice. **(D)** Western blot analysis demonstrated that α2δ1 expression was reduced in tumor tissues injected with AAV zy9-3/shα2δ1 after a two-week period (n = 4; β-Actin was employed as the loading control). ** indicates P < 0.01, *** indicates P < 0.001.

All eight nude mice inoculated with 2 × 10^5^ SCC-090 cells developed subcutaneous tumor nodules of similar size on day 6 after inoculation of the tumor cells. Each mouse was administered an intravenous injection of 10^12^ vg of AAVzy9-3/shα2δ1 (expressing iRFP) or an equivalent volume of phosphate buffered saline (PBS; n = 4) and photographed and tumor volume and size measured 2 weeks after injection. The tumor tissues in the AAV-treated group exhibited robust iRFP expression, whereas no iRFP expression was observed in other tissues and organs. In contrast, the tumors in the PBS group were markedly larger in size compared to those in the AAV-treated group (**Figure 6A**). The tumor volume in the PBS group was greater than that in the treated group (**Figure 6B**), and similarly, the mass of the PBS group was significantly higher than that of AAV-treated group (**Figure 6C**). Extraction of protein from each mouse tumor tissue showed a significant knockdown of α2δ1 (**Figure 6D**). These results demonstrate the efficacy of AAVzy9–3 as a vector for the treatment of HNSCC.

## Discussion

The scientific literature on cancer research demonstrates that cancer is a disease characterized by dynamic changes in the genome (10). Among these, head and neck tumors can be treated with intratumoral injections and are amenable to gene therapy trials (12). Genomic sequencing of tumor biopsies has transformed cancer care and drug development (23).

The success of tumor gene therapy depends on delivery vectors, categorized as viral or non-viral. Non-viral systems, including polymers, lipid nanoparticles (LNPs), and inorganic hybrids, have gained prominence due to their combined advantages (24). Compared to viral vectors, non-viral platforms exhibit lower risks of cytotoxicity, immunogenicity, and mutagenesis risks, driving their clinical exploration (25, 26). LNPs exemplify this progress, achieving clinical translation for siRNA, mRNA, and small molecule deliver (27). In particular, Yuebao Zhang et al. (28) demonstrated that integration of lipid nanoparticle-mRNA formulations and dendritic cell therapy can close the cancer-immunity cycle. However, challenges remain in terms of efficiency, specificity, sustained expression, and long-term safety (24). Contemporary viral vector-based gene therapy is achieved by *in vivo* delivery of the therapeutic gene into the patient by vectors based on retroviruses, Ads or AAVs (29). Retroviruses excel at integrating exogenous genes into the host genome for long-term stable expression and can carry larger gene fragments than AAVs, with higher transfection efficiency in dividing cells (30). However, their random genomic integration can activate proto-oncogenes or inactivate tumor suppressor genes, with uncertain effects (31). In addition, retroviruses are restricted to infecting dividing cells, are prone to triggering host immune responses, and, like AAV vectors, are expensive to produce in large quantities (32).

The AAV vectors represent a delivery system that has been successfully used in both basic and preclinical studies as well as in clinical applications, particularly *in vivo (* 33). Nevertheless, the primary challenge for AAV-mediated gene therapy is to enhance the transduction and expression efficiency in target tissues (34). The AAV are classified into different serotypes according to the amino acid sequences of their coat proteins and the length of their genomes (35). The different serotypes have varying cell attachment sites and entry modes, and efficient vectors can be tailored to target different tissues by engineering modifications of the capsid (36). Among these approaches, the DNA shuffled vector library-based approach does not necessitate detailed knowledge of the coat structure, which is random but more straightforward to manipulate (37). The idea behind gene shuffling libraries is to combine multiple advantageous features of different AAV serotypes (38). However, this approach is time-consuming because of the many variants and the *in vivo* selection process (39). In this study, we constructed a new AAV capsid library using a directed evolutionary approach based on random point mutation and chimeric capsid scaffolds and performed multiple rounds of selection in cell culture to screen for new AAV capsids capable of efficiently transducing HNSCC. This was followed by multiple rounds of selection in cell culture, with the aim of screening for new AAV capsids capable of efficiently transducing HNSCC. This strategy yielded novel AAV capsids that exhibited enhanced transduction efficiency in SCC-090 and other HNSCC cell lines, as compared to the parental serotypes. Furthermore, these capsids demonstrated superior targeting and infectivity *in vivo*.

Given the limited research on HNSCC-associated AAV vectors, we chose 12 serotypes as parents for diversity. Random point mutations were introduced based on DNA shuffling. The technology for targeted AAV capsid evolution is nearly mature. Franz Schweiggert et al. (40) developed a tool to analyze and visualize the diversity of AAV DNA shuffling libraries and the composition of final variants. Researchers now focus more on *in vivo* screening due to the limitations of *in vitro* AAV capsid library screening, i.e., *in vivo* application may not be as effective as *in vitro* application. We addressed this by screening various HNSCC cells and found that the AAVzy9–3 capsid had the best targeting properties and was effective in infecting other HNSCC cells. This capsid is groundbreaking as it’s the first to target HNSCC. Minghong Jiang et al. (12) used wild-type AAV2 in the treatment of HNSCC using AAV vectors. Interestingly, the second half of VP3 of our AAVzy9–3 is completely derived from AAV2. Jun Wang et al. (41) showed that a hybrid baculovirus-adeno-associated virus (BV-AAV) could modify bone marrow mesenchymal stem cells to express the sodium iodide symporter, facilitating radioiodine therapy for hypopharyngeal carcinoma, reducing tumor growth, and increasing survival. Further testing of systemic and local administration modes is expected to improve therapeutic effects. Additionally, recent studies on enhancing AAV vector infectivity and targeting through directed evolution and rational design (insertion of peptides with specific targeting properties at specific sites (11, 42)) will be incorporated into our future research, potentially combined with HNSCC or tumor cell-targeting promoters for gene therapy.

The HNSCC is associated with a poor prognosis, with a 5-year survival rate of only 25–60% following treatment (43). Our previous research has previously confirmed α2δ1 as a specific cancer stem cell marker in laryngeal squamous carcinoma, with α2δ1-positive cells showing higher self-renewal, invasion and migration abilities. α2δ1 has been demonstrated to mediate their stem cell properties, chemotherapy tolerance and radiotherapy resistance (44). Encoded by CACNA2D1, α2δ1 is a crucial component of L-type voltage-gated calcium channels, widely expressed in human cells (45). Our previous study used lentiviral ectopic overexpression and shRNA to knock down α2δ1, but this method had limitations *in vivo*. Using an HNSCC-targeted AAV vector, we achieved effective intratumoral knockdown without adverse health effects in mice.

In summary, we present a pioneering AAV capsid variant with augmented HNSCC targeting capabilities. The data presented herein demonstrate the therapeutic potential of the vector in mouse models, characterized by enhanced targeting and infection efficiency. This evolved AAV variant is expected to reduce the required doses for HNSCC gene therapy, and offer significant advantages for clinical translation, including optimized delivery and improved safety profiles.

## Ethics approval

This study was performed in line with the principles of the Declaration of Helsinki. Approval was granted by the Ethics Committee of Beijing Friendship Hospital, Capital Medical University (No. 2023-P2-045-01)

## Data Availability

The data that support the findings of this study have been deposited into CNGB Sequence Archive (CNSA) of China National GeneBank DataBase (CNGBdb) with accession number CNP0007138 (https://db.cngb.org/search/project/CNP0007138/).
