# Supplementary material for: Adeno-associated virus vector modification based on directed evolution technology for gene therapy targeting head and neck squamous cell carcinoma
Source: Front Oncol. 2025 Jun 3;15:1566584. doi: 10.3389/fonc.2025.1566584 (PMC12170314; doi:10.3389/fonc.2025.1566584)
Supplement: Supplementary file 1 [file DataSheet1.docx]

**Supplemental Methods:**

**shRNA constructs**

The shRNAs were cloned into pAAV-shRNA-CMV-iRFP containing an H1 promoter (the plasmid was synthesized by Tsingke Biotechnology Co., Ltd.) flanked with BamHⅠ and EcoRⅠrestriction sites. The plasmid was digested with BamHⅠ (New England Biolabs, R3138L) and EcoRⅠ (New England Biolabs, R3101L). Three shRNA sequences targeting the human α2δ1 gene (NM_000722.4) were designed using online design tools. Oligonucleotides targeting α2δ1 or a non-specific target (for the α2δ1 shRNA -1# - forward oligo 5' GATCCGCTGAAGATCATGCAGGATTATTCAAGAGATAATCCTGCATGATCTTCAGCTTTTTG 3', reverse oligo 5' AATTCAAAAATGAAAGATCATGCAGGATTAATCTCTTGAAGATGTCAGTGGCATCGTCTTTCAG 3', for the α2δ1 shRNA -2# - forward oligo 5' GATCCGCACGCCAGCTGGTAGAAATTCTTCCTGTCAGAAATTTCTACCAGCTGGCGTGCTTTTTG 3', reverse oligo 5' AATTCAAAAAGCACGCCAGCTGGTAGAAATTTCTGACAGGAAGAATTTCTACCAGCTGGCGTGCG 3', or the α2δ1 shRNA -3# - forward oligo 5' GATCCGCAATGAAGTTGTCTACTACACTTCCTGTCAGATGTAGTAGACAACTTCATTGCTTTTTG 3', reverse oligo 5' AATTCAAAAAGCAATGAAGTTGTCTACTACATCTGACAGGAAGTGTAGTAGACAACTTCATTGCG 3', and non-specific control - forward oligo 5' GATCCTGAAAGACGATGCCACTGACACTTCCTGTCAGATGTCAGTGGCATCGTCTTTCATTTTTG 3', reverse oligo 5' AATTCAAAAATGAAAGACGATGCCACTGACATCTGACAGGAAGTGTCAGTGGCATCGTCTTTCAG 3') were annealed, resulting in a fragment containing a 5' BamHⅠ restriction site and a 3' EcoRⅠ restriction site overhang, and cloned into the restriction digested plasmid, to generate pAAV-shα2δ1-1#, pAAV-shα2δ1-2#, pAAV-shα2δ1-3#, and pAAV-shRNA-Ctrl.

**AAV production and *in vitro* validation of α2δ1 knockdown in SCC-090 cells**

**AAV production by triple plasmid co-transfection. AAVzy9-3, pPLUS® AAV-Helper (Polyplus, 101000183) and pAAV-shα2δ1 plasmids (1#, 2#, 3#) or control plasmid (pAAV-shRNA-Ctrl,** pAAV-shRNA-CMV-iRFP**) were used. The three plasmids were co-transfected into 2×10^5^ 239T cells using FectoVIR® AAV (Polyplus, 101000044) in an equimolar ratio. At 72 h post-transfection, viral particles were harvested and purified through a preformed discontinuous iodixanol gradient of 15%, 25%, 40%, 60% iodixanol (OptiPrep, 1893) by ultracentrifugation (160000 ×g, 4°C, 2 h).** This purification process yielded the AAVzy9-3/shα2δ1 (1#, 2#, 3#), AAVzy9-3/shRNA-Ctrl and empty viruses. **Viral genome titers were then quantified by qPCR using AAV2 rep-specific primers.**

SCC-090 cells were seeded in 6-well plates at a density of 5 × 10⁵ cells per well. After 24 hours of incubation in a cell culture incubator, the medium was changed. The culture medium was then replaced with fresh medium containing one of five AAV vectors per well: AAVzy9-3/shα2δ1-1#, AAVzy9-3/shα2δ1-2#, AAVzy9-3/shα2δ1-3#, and control group: AAVzy9-3/shRNA-Ctrl and empty vector, were added to separate wells at an MOI of 10⁴. The cells were then incubated in a cell culture incubator at 37°C with 5% CO₂ for 72 h. The cell supernatant was discarded, and proteins were extracted from the cells in each well after rinsing twice with PBS, and the protein expression levels were verified by Western blot.

**Supplemental result:**

**AAV-mediated shRNA knockdown of α2δ1 in SCC-090 cells**

Knockdown of transcripts using small hairpin RNA (shRNA) is a powerful tool for studying gene function. Three shRNAs targeting α2δ1 were designed and tested in SCC-090 cells. A non-specific shRNA and an empty vector were each designed and used as controls for shRNA transduction.The knockdown efficiency of α2δ1 expression mediated by Western blot analysis confirmed effective α2δ1 knockdown (reduced expression) mediated by the AAVzy9-3 vector in SCC-090 cells (Fig S1). Negative controls (including non-specific shRNA and empty vector groups) showed no detectable reduction in α2δ1 levels. Grayscale quantification demonstrated that both shRNA constructs targeting sites 2# and 3# achieved significant α2δ1 knockdown compared to controls (Fig S1B). Although statistical analysis revealed no significant difference in knockdown efficiency between the two sites, site 2# displayed a marginally stronger reduction in α2δ1 protein levels by qualitative visual assessment. Based on this observed trend toward improved efficacy, site 2# was selected for subsequent in vivo therapeutic studies.

**
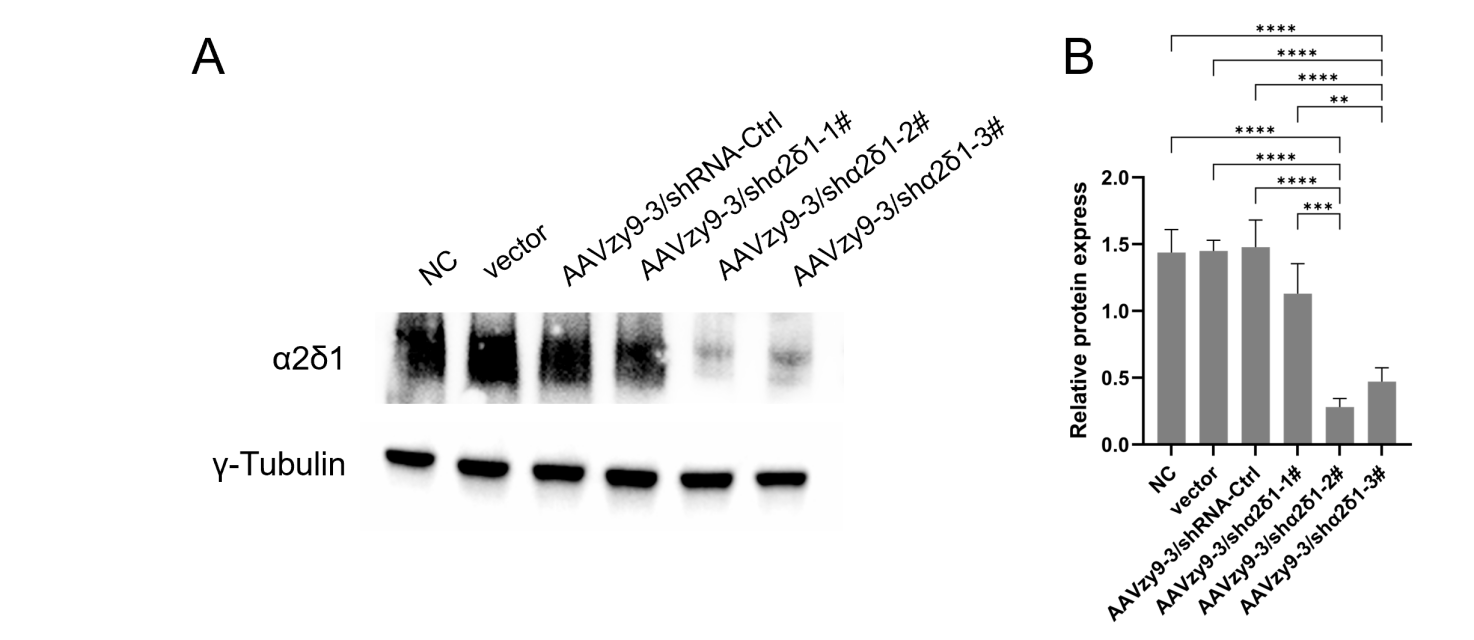
**

****Fig. S1** (A) Western blot analysis of α2δ1 knockdown in SCC-090 cells. (B) Densitometric analysis of α2δ1 knockdown efficiency.**
