# Supplementary material for: Adeno-associated virus vector modification based on directed evolution technology for gene therapy targeting head and neck squamous cell carcinoma
Source: Front Oncol. 2025 Jun 3;15:1566584. doi: 10.3389/fonc.2025.1566584 (PMC12170314; doi:10.3389/fonc.2025.1566584)
Supplement: Supplementary file 2 [file Table1.docx]

The specific sequence of the *cap* gene of AAVzy9-3 is given in the Supplementary Material.

The original contributions presented in the study are publicly available. The data that support the findings of this study have been deposited into CNGB Sequence Archive (CNSA) of China National GeneBank DataBase (CNGBdb) with accession number CNP0007138 (https://db.cngb.org/search/project/CNP0007138/).

The specific sequence is as follows:

5’-ATGGCTGCCGATGGTTATCTTCCAGATTGGCTCGAGGACAACCTTTCTGAAGGCATTCGTGAGTGGTGGGCTCTGAAACCTGGAGTCCCTCAACCCAAAGCGAACCAACAACACCAGGACAACCGTCGGGGTCTTGTGCTTCCGGGTTACAAATACCTCGGACCCGGTAACGGACTCGACAAAGGGGAGCCGGTCAACGCAGCAGACGCGGCAGCCCTCGAACACGACAAAGCTTACGACCAGCAGCTCAAGGCCGGTGACAACCCGTACCTCAAGTACAACCACGCCGACGCCGAGTTTCAGGAGCGTCTTCAAGAAGATACGTCTTTTGGGGGCAACCTTGGCAGAGCAGTCTTCCAGGCCAAAAAGAGGATCCTTGAGCCTCTTGGTCTGGTTGAGGAAGCAGCTAAAACGGCTCCTGGAAAGAAGAGGCCTGTAGATCAGTCTCCTCAGGAACCGGACTCATCATCTGGTGTTGGCAAATCGGGCAAACAGCCTGCCAGAAAAAGACTAAATTTCGGTCAGACTGGCGACTCAGAGTCAGTCCCCGACCCTCAACCTCTCGGAGAACCTCCAGCAGCGCCCTCTAGTGTGGGATCTGGTACAGTGGCTGCAGGCGGTGGCGCACCAATGGCAGACAATAACGAAGGTGCCGACGGAGTGGGTAATGCCTCAGGAAATTGGCATTGCGATTCCACATGGCTGGGCGACAGAGTCATTACCACCAGCACCCGAACCTGGGCCCTGCCCACCTACAACAACCACCTCTACAAGCAAATCTCCAGTGAAACTGCAGGTAGTACCAACGACAACACCTACTTCGGCTACAGCACCCCCTGGGGGTATTTTGACTTTAACAGATTCCACTGCCACTTCTCACCACGTGACTGGCAGCGACTCATCAACAACAACTGGGGATTCCGGCCCAAGAAGCTGCGGTTCAAGCTCTTCAACATCCAGGTCAAGGAGGTCACGACGAATGACGGCGTTACGACCATCGCTAATAACCTTACCAGCACGGTTCAAGTCTTCTCGGACTCGGAGTACCAGCTTCCGTACGTCCTCGGCTCTGCGCACCAGGGCTGCCTCCCTCCGTTCCCGGCGGACGTGTTCATGATTCCGCAATACGGCTACCTGACGCTCAACAATGGCAGCCAAGCCGTGGGACGTTCATCCTTTTACTGCCTGGAATATTTCCCTTCTCAGATGCTGAGAACGGGCAACAACTTTACCTTCAGCTACACCTTCGAGGACGTGCCTTTCCACAGCAGCTACGCTCACAGCCAGAGTCTGGACCGTCTCATGAATCCTCTCATCGACCAGTACCTGTATTACTTGAGCAGAACAAACACTCCAAGTGGAACCACCACGCAGTCAAGGCTTCAGTTTTCTCAGGCCGGAGCGAGTGACATTCGGGACCAGTCTAGGAACTGGCTTCCTGGACCCTGTTACCGCCAGCAGCGAGTATCAAAGACATCTGCGGATAACAACAACAGTGAATACTCGTGGACTGGAGCTACCAAGTACCACCTCAATGGCAGAGACTCTCTGGTGAATCCGGGCCCGGCCATGGCAAGCCACAAGGACGATGAAGAAAAGTTTTTTCCTCAGAGCGGGGTTCTCATCTTTGGGAAGCAAGGCTCAGAGAAAACAAATGTGGACATTGAAAAGGTCATGATTACAGACGAAGAGGAAATCAGGACAACCAATCCCGTGGCTACGGAGCAGTATGGTTCTGTATCTACCAACCTCCAGAGAGGCAACAGACAAGCAGCTACCGCAGATGTCAACACACAAGGCGTTCTTCCAGGCATGGTCTGGCAGGACAGAGATGTGTACCTTCAGGGGCCCATCTGGGCAAAGATTCCACACACGGACGGACATTTTCACCCCTCTCCCCTCATGGGTGGATTCGGACTTAAACACCCTCCTCCACAGATTCTCATCAAGAACACCCCGGTACCTGCGAATCCTTCGACCACCTTCAGTGCGGCAAAGTTTGCTTCCTTCATCACACAGTACTCCACGGGACAGGTCAGCGTGGAGATCGAGTGGGAGCTGCAGAAGGAAAACAGCAAACGCTGGAATCCCGAAATTCAGTACACTTCCAACTACAACAAGTCTGTTAATGTGGACTTTACTGTGGACACTAATGGCGTGTATTCAGAGCCTCGCCCCATTGGCACCAGATACCTGACTCGTAATCTGTAA-3’
